# eCALIBRATOR: A Comparative Tool to Identify Key Genes and Pathways for *Eucalyptus* Defense Against Biotic Stressors

**DOI:** 10.3389/fmicb.2020.00216

**Published:** 2020-02-17

**Authors:** Yves du Toit, Donovin William Coles, Ritesh Mewalal, Nanette Christie, Sanushka Naidoo

**Affiliations:** ^1^Department of Biochemistry, Genetics and Microbiology, Forestry and Agricultural Biotechnology Institute (FABI), University of Pretoria, Pretoria, South Africa; ^2^Environmental Genomics and Systems Biology Division, Lawrence Berkeley National Laboratory, DOE Joint Genome Institute, Berkeley, CA, United States

**Keywords:** *Eucalyptus*, comparative transcriptomics, nitrogen, nitrate transporter, defense

## Abstract

Many pests and pathogens threaten Eucalyptus plantations. The study of defense responses in this economically important wood and fiber crop enables the discovery of novel pathways and genes, which may be adopted to improve resistance. Various functional genomics experiments have been conducted in Eucalyptus-biotic stress interactions following the availability of the *Eucalyptus grandis* genome, however, comparisons between these studies were limited largely due to a lack of comparative tools. To this end, we developed eCALIBRATOR http://ecalibrator.bi.up.ac.za, a tool for the comparison of Eucalyptus biotic stress interaction. The tool, which is not limited to Eucalyptus, allows the comparison of various datasets, provides a visual output in the form of Venn diagrams and clustering and extraction of lists for gene ontology enrichment analyses. We also demonstrate the usefulness of the tool in revealing pathways and key gene targets to further functionally characterize. We identified 708 differentially expressed *E. grandis* genes in common among responses to the insect pest *Leptocybe invasa*, oomycete pathogen *Phytophthora cinnamomi* and fungus *Chrysoporthe austroafricana*. Within this set of genes, one of the Gene Ontology terms enriched was “response to organonitrogen compound,” with *NITRATE TRANSPORTER 2.5* (*NRT2.5*) being a key gene, up-regulated under susceptible interactions and down-regulated under resistant interactions. Although previous functional genetics studies in *Arabidopsis thaliana* support a role in nitrate acquisition and remobilization under long-term nitrate starvation, the importance of *NRT2.5* in plant defense is unclear. The T-DNA mutants of *AtNRT2.5* were more resistant to *Pseudomonas syringae* pv. *tomato pv tomato* DC3000 inoculation than the wild-type counterpart, supporting a direct role for *NRT2.5* in plant defense. Future studies will focus on characterizing the *Eucalyptus* ortholog of *NRT2.5*.

## Introduction

Eucalyptus species and hybrids are threatened by various pests and pathogens. This is largely owing to the extradition of the species to other parts of the world. Outside of its native range where natural enemies are not present, the pests and pathogens proliferate. In recent years, clonal forestry is favored for improved productivity but such forestry is in danger of being decimated by a single pathogen or pest if the selected tree genotype is susceptible (Wingfield et al., 2008).

Some important pests on *Eucalyptus* are the insect pest, *Leptocybe invasa*, which causes galls in young leaves, and *Gonipterus scutellatus* (bronze bug), which causes damage to leaves through feeding (Wingfield et al., 2008). The pathogens which are considered major challenges to Eucalyptus species include *Austropuccinia psidii, Teratosphaeria destructans, Teratosphaeria zuluense, Ceratocystis fimbriata, Chrysoporthe austroafricana*, and *Phytophthora cinnamomi* [reviewed in Wingfield et al. (2008) and Maússe-Sitoe et al. (2016); Wingfield MJ personal communication]. The female *L. invasa* wasp, causes the formation of galls when ovipositing on leaf and stem tissues, laying eggs in the upper epidermal layer of young *Eucalyptus* leaves (Mendel et al., 2004). Typical symptoms of *Eucalyptus* leaf and stem attack by *L. invasa* include, bump-shaped galls on the leaf midribs, petioles and stems of several *Eucalyptus* species and clones (Neser et al., 2007). Infection by *C. austroafricana* results in canker formation near the base of the tree (Heath et al., 2006), which is followed by the rapid wilt and death of the tree, if the tree is young (Roux et al., 2003). On mature trees, the bark appears cracked near the base of the tree, which renders these trees susceptible to being blown over by strong winds (Rodas et al., 2005; Nakabonge et al., 2006). Symptoms of infection by *P. cinnamomi* are root rot and stem cankers which often results in die-back (Hardy et al., 1996; Hardham, 2005). This is believed to be due to the lack of water movement through the process of transpiration. Knowledge of the defense responses induced upon the pest or pathogen challenge can aid in the development of strategies to improve tree defenses and select for more resistant tree genotypes (Naidoo et al., 2019).

Previous studies conducted RNA-sequencing on some of the above-mentioned Eucalyptus biotic stress interactions. These included *Eucalyptus grandis* and *L. invasa* (Oates et al., 2015), *E. grandis* and *C. austroafricana* (Mangwanda et al., 2015), and *Eucalyptus nitens* and *Phytophthora cinnamomi* (Meyer et al., 2016). In response to *P. cinnamomi*, Meyer et al. (2016) identified sets of Eucalyptus differentially expressed genes enriched in various Gene Ontologies (GO) for Biological Processes (BP). These included jasmonic acid and ethylene signaling, phenylpropanoid pathway, aromatic compound synthesis terms and terms relating to the response to water deprivation and water stress in the up-regulated gene set. While terms generally relating to growth, cell wall modifications, cell wall chemistry, the phenylpropanoid pathway, auxin and gibberellin hormones as well as photosynthesis were observed in the down-regulated gene set (Meyer et al., 2016). In the response to *C. austroafricana*, Mangwanda et al. (2015) found GO BP terms associated with the phenylpropanoid pathway, response to oxidative stress, secondary metabolic process, lignin metabolic processes, response to ethylene and jasmonic acid were apparent in the up-regulated gene set. Interestingly the down-regulated gene set consisted of enriched terms relating to photosynthesis related processes (Mangwanda et al., 2015). In the *E. grandis* – *L. invasa* interaction, Oates et al. (2015) identified a set of enriched GO BP terms related to the terpenoid biosynthesis process as well as the flavonoid biosynthesis process.

Comparative transcriptomic analyses using compatible and incompatible interactions of multiple pathogen interactions with the same host, is valuable to reveal shared and tailored defense responses. A classic example is the study of De Vos et al. (2005) where various *Arabidopsis thaliana* – attacker combinations were compared. This included the leaf bacteria *Pseudomonas syringae* pv. *tomato*, a leaf pathogenic fungus *Alternaria brassicicola*, the chewing caterpillar, *Pieris rapae*, feeding thrips, *Frankliniella occidentalis* and aphid *Myzus persicae*. Results of these comparisons suggested that salicylic acid (SA), jasmonic acid (JA), and ethylene (ET), played an important role in shared *A. thaliana* defenses. However, defense responses to specific pathogens were tailored through regulatory mechanisms. This could be pathway cross-talk or the response to specific attacker induced signaling (De Vos et al., 2005).

Although the defense responses of these three *Eucalyptus –* biotic stress interactions were individually investigated by comparing the transcriptomes of mock inoculated and inoculated samples of compatible and incompatible interactions using *Eucalyptus* clones, comparisons between these responses were not performed; this was largely due to a lack of user friendly bioinformatic tools to conduct such analyses.

The aim of this study was to (i) develop interactive tools that will facilitate comparative transcriptomic analysis, (ii) apply these tools to interrogate and identify important defense response pathways and genes of Eucalyptus under biotic stress challenge, and (iii) subject one of the targets for functional testing to verify the usefulness of the tool. We developed eCALIBRATOR – a tool to compare Eucalyptus defense responses for the downstream selection of common pathways and genes that have important implications for defense in Eucalyptus. We also demonstrate the usefulness of the tool by identifying nitrate transporter 2.5 (NRT2.5) as a key target for functional characterization and go on to test the Arabidopsis knock-out. Our work shows that AtNRT2.5 is implicated in plant defense.

## Materials and Methods

### RNA-Seq Libraries

The RNA-seq was previously conducted for plant pathogen interactions involving Eucalyptus and *C. austroafricana*, *P. cinammomi*, and *L. invasa*, respectively (Mangwanda et al., 2015; Oates et al., 2015; Meyer et al., 2016). For the interactions involving *L. invasa* and *C. austroafricana*, RNA-seq libraries were sequenced for moderately resistant and susceptible clones, whereas only the susceptible interaction was sequenced for the interaction involving *P. cinnamomi*. Together, there were five experimental genotypes (Table 1). The resistant and susceptible clones were composed of treated and untreated samples. Each sample was composed of three biological replicates, making a total of 30 RNA-seq libraries.

**TABLE 1:** Summary of expressed and differentially expressed genes for each of the Eucalyptus interactions and tissue comparisons.

	Plant pathogein interaction		Expreesed genes		Differentialy expressed genes		
Tissue	Interacting pathogen	Resistant/susceptible (Host)	Control	Treated	Tissue comparison	Interaction comparison	Resistance comparison
Leaf	L *invasa*	Resistant (E. *grandis* TAG5)	28 603	28 434	8 610	8 610	5 410
	*L invasa*	Susceptible (E. *grandis* GC540)	29 680	29 462			5 846
Stem	*C. austroafricona*	Resistant (E. *grandis* TAG5)	28 268	28 499	15 699	11854	8 958
	*C. austroafricona*	Susceptible (E. *grandis* ZG14)	28 268	28 499			8 304
	*P. cinnamomi*	Susceptible (E. *nitens)*	27 920	27 774		8 414	9 414

### RNA-Seq Read Mapping and Expression Analysis

For each RNA-seq library, FASTQC v0.52 was used to verify RNA-seq data quality. The 30 libraries were then subjected to expression analysis using the Tuxedo suite (Trapnell et al., 2012). Short (50 bp) forward and reverse reads were aligned and mapped to the *E. grandis* genome v1.1 using Tophat v2.0.14 to identify genes and their isoforms. Cufflinks v2.2.1 was used to identify and quantify gene and transcript expression, using previously defined gene and transcript models. Quantification of gene and transcript expression was done using FPKM (Fragments Per Kilo-base of exon model per Million mapped fragments) as the measure of expression. Quartile normalization and bias correction was performed for each sample. Finally, sets of differentially expressed genes were identified using Cuffdiff v2.2.1 (Trapnell et al., 2012). The treated samples were compared to their equivalent mock-inoculated samples and the log2 fold-change [log2(average FPKM: treated/average FPKM: control)] was determined for each gene and transcript, respectively, after which a *p*-value was then calculated (Trapnell et al., 2010). The Benjamini–Hochberg multiple test correction was performed to adjust the *p*-values by controlling the false discovery rate (Benjamini and Hochberg, 1995).

### Transcriptomic Comparisons

The 30 RNA-seq libraries were converted through differential expression analysis into five gene lists (one for each of the five genotypes tested); which were quantified with a corresponding log2 fold-change value, calculated per gene. The five gene lists were used in three transcriptomic comparisons to understand the shared and tailored Eucalyptus defenses under different biotic stress challenges (Table 1). The “tissue comparison,” compared the leaf tissue interaction transcriptomes (two gene lists) with the stem tissue interaction transcriptomes (three gene lists). This comparison was used to identify tissue specific defense responses under different biotic challenges in Eucalyptus. After identifying all significantly differentially expressed genes (*q*-value < 0.05) within each of the five datasets, the two leaf datasets were merged into a leaf representative dataset by combining the gene lists and removing the duplicates. A similar strategy was conducted for the three stem datasets. The “interaction comparison” compared the transcriptomes of the *L. invasa* interaction (two gene lists), the *C. austroafricana* interaction (two gene lists), and the *P. cinnamomi* interaction (one gene list). This comparison was used to identify Eucalyptus defense responses that were shared or found to be unique, when Eucalyptus was challenged by different biotic stresses. After identifying all significantly differentially expressed genes (*q*-value < 0.05) within each of the five datasets, the datasets making up each interaction were merged into interaction representative datasets by combining the gene lists and removing the duplicates. The “resistance comparison” was conducted between the moderately resistant and susceptible interactions per pathogen interaction with *Eucalyptus.* This comparison was used to identify aspects of defense that are related to *Eucalyptus* resistance against specific biotic stress challenges, and aspects of defense that were shared among biotic stress challenges. The significantly differentially expressed genes (*q*-value < 0.05) within each of the five datasets were used.

### The Eucalyptus Interactive Biotic Stress Investigator (eCALIBRATOR)

eCALIBRATOR is a web-based tool that was coded using a combination of web-based programming languages (Hypertext Markup Language (HTML), cascading style sheets (CSS) and Javascript). eCALIBRATOR uses hypertext pre-processor (PHP) queries to retrieve information from the accompanying Eucalyptus Biotic Stress database, containing information relating to the three Eucalyptus biotic stress interactions mentioned above. The database was created through the MySQL relational database management system and can be accessed through the eCALIBRATOR web-based tool via the “data browse” option. eCALIBRATOR consists of two comparative and interactive exploratory tools namely VennPlot and HCPlot, which can be utilized to compare transcriptomic datasets. The VennPlot, based on jvenn (Bardou et al., 2014), accepts up to six gene lists, which are either uploaded by the investigator, or retrieved from the database. The VennPlot tool identifies common and unique genes by calculating all intersects between the submitted datasets. VennPlot reports an interactive Venn or Edwards diagram; as well as providing the respective gene lists for all calculated intercepts. The HCPlot tool, using InCHlib (Škuta et al., 2014), identifies patterns of expression by grouping sets of genes that show similar patterns of expression through a process of hierarchical clustering. eCALIBRATOR can be feely accessed at http://ecalibrator.bi.up.ac.za.

### Hierarchical Clustering

To generate the results for the transcriptomic comparisons mentioned above, hierarchical clustering was performed on the subset of genes for which it was possible to calculate log2 fold-change values (genes that were expressed in both treated and untreated samples). Spearman correlation was used as a distance metric, together with the complete agglomeration algorithm. The number of clusters was determined by dividing the dendrogram height by 5. Hierarchical clustering was conducted in R (v3.4.1) using the hclust package.

### Gene Ontology Enrichment

Functional enrichment was performed with the TopGO (v2.28.0) package (Alexa and Rahnenfuhrer, 2010) in R. The Elim algorithm was used to reduce local dependency and redundancy in enriched GO terms in the biological process category. Enrichment was performed with the *E. grandis* genome v1.1 as the gene universe, and the gene-to-GO map file that was used was created by Kersting et al. (2015). The TopGO parameters that were changed from the default values were: topNodes = 50; nodeSize = 5. A *p*-value was calculated with the Fisher’s exact test with an adjustment for multiple testing using the false discovery rate.

### Phylogenetic Analysis of NRT2.5 and Gene Family Members

The amino acid sequences of *A. thaliana*, *E. grandis*, and *Populus trichocarpa* NRT2.5 and family members were obtained from Phytozome v10. To achieve this, the AtNRT2.5 genomic sequence was used in a BLAST search against the proteome of *A. thaliana* TAIR10 to obtain the amino acid sequences of AtNRT2.5 and all other family members (AtNRT2.1-AtNRT2.7). Once obtained, the amino acid sequences of AtNRT2.5 and all family members were used in a BLAST search against the proteome of both *E. grandis* and *P. trichocarpa* to obtain the NRT2 amino acid sequences in these species. The Expected (E) threshold was set to −1. Once all amino acid sequences were obtained, a reciprocal BLAST using each *E. grandis* sequence was performed against the proteome of *A. thaliana* to identify one-to-one orthologs. The amino acid sequences of the NRT2 family from *A. thaliana*, *E. grandis*, and *P. trichocarpa* were then used to generate a phylogenetic tree using Saté-II^1^. The SATé-II-simple variant achieved the best score, therefore the alignment from this variant was used for further analyses. The low quality sequences from this alignment were trimmed using trimAl^2^ through command line. The trimmed sequences were then imported into winSCP^3^ and FastTree2 was used to construct the maximum-likelihood phylogenetic tree through PuTTY^4^ with default parameters.

### Generation of Homozygous T-DNA Insertion Lines

Transgenic generation 3 (T3) seeds of two *atnrt2.5* T-DNA insertion mutants, GABI-Kat 213H10 (*atnrt2.5-A*), provided by the Nottingham *Arabidopsis* Stock Centre (Kleinboelting et al., 2012) and GABI-Kat 046H04 (*atnrt2.5-B*), provided by GABI-Kat (Scholl et al., 2000) in Col-0 background were used to screen for homozygotes. Positive lines were selected on Murashige and Skoog (MS) media supplemented with sulfadiazine sodium salt (7.5 mg. mL^–1^). Transgenic selection was carried out on medium containing 7.5 mg/ml sulfadiazine sodium salt (Sigma-Aldrich) or 20 μg/ml Hygromycin B (Roche, Mannheim, Germany). Homozygous T-DNA lines were identified by PCR genotyping using a combination of the T-DNA left border oligonucleotide and gene-specific oligonucleotides. Homozygous lines were confirmed on sulfadiazine MS plates, using a chi-squared test (Cochran, 1952).

### Plant Growth Conditions

Arabidopsis seeds of Columbia (Col-0) ecotype and homozygous T3 mutant seeds were surface sterilized (10% bleach and 0.1% triton-X100 solution) before sowing on 0.5x MS agar plates (MS; pH 5.9, 0.8% Bacto agar, Merck) or modified germination medium (1 mM KNO_3_, 0.8% Bacto agar). Seeds were stratified for 2 days at 4°C, then transferred to an IPS750 incubator (Memmert, Schwabach, Germany) at ∼22°C for 2 weeks under long day conditions (16 h day and 8 h night). Seedlings were transplanted in peat moss bags (Jiffy^TM^ Products International AS, Kristiansand, Norway) and grown in controlled growth rooms under 10 h of light per 24 h at ∼22°C and 60% relative humidity with optimal fertilization of 7 mM nitrate.

### Bacterial Proliferation Assay and Disease Scoring

We used the well-established pathosystem of Arabidopsis and *Pseudomonas syringae* pv. *tomato* DC3000 to determine if NRT2.5 was involved in plant defense (Saleem et al., 2017). The bacterial strain *Pst* DC3000 was kindly donated by Dr. Shane Murray (Department of Molecular and Cell Biology, University of Cape Town). Bacterial inoculum was prepared according to the method by Tornero and Dangl (2001). The bacteria were grown on King’s B media (2% protease peptone, 1.5% Bacto agar, 1% glycerol, 0.15% K_2_HPO_4_, 0.15% MgSO_4_, pH 7.2; King et al., 1954) supplemented with rifampicin 50 μg/mL for 24 h at 28°C and the bacterial suspension was adjusted to an OD_600_ of 0.05 using 10 mM MgCl_2_. Arabidopsis wild-type and mutant plants of 5-weeks-old (or otherwise mentioned) were inoculated by dipping the rosette in the bacterial suspension using Silwet L-77 at a final concentration of 0.02% for 10 s and thereafter enclosed with a transparent lid to create high humidity for 1 h. Mock-inoculated plants were dipped in a solution of 10 mM MgCl_2_ and Silwet L-77 0.02% without bacteria. Bacterial growth was determined in wild-type and mutant plants by extracting bacteria and calculating the number of rifampicin resistant colony forming units per leaf disk over a 3-day time interval (Liu et al., 2015). Three days after inoculation, the percentage of diseased leaves was determined for each plant (*n* = 20–25) (Camañes et al., 2012). Leaves were scored as diseased if characteristic symptoms including necrosis and water-soaked lesions surrounded by chlorosis were observed. Statistical analysis of data was performed using ANOVA and a Least Significant Difference (LSD) test.

## Results

### RNA-Seq Expression Data

RNA-seq of the *L. invasa, C. austroafricana*, and *P. cinnamomi* interactions with resistant and susceptible Eucalyptus hosts, resulted in 30 RNA-seq libraries. Supplementary Table S1 depicts the number of reads per biological replicate per sample, for each genotype of each interaction. On average, the concordant mapping rate was 80.26%. The average number of expressed genes across the three biological replicates and the number of differentially expressed genes per interaction is given in Table 1. *P. cinnamomi* had the most differentially expressed genes overall, followed by *C. austroafricana*, and then *L. invasa*. Interestingly there were less differentially expressed genes in the moderately resistant interaction with *L. invasa* and more differentially expressed genes in the *C. austroafricana* interaction.

### The Eucalyptus Interactive Biotic Stress Investigator (eCALIBRATOR) Web-Based Tool

**FIGURE 1:**
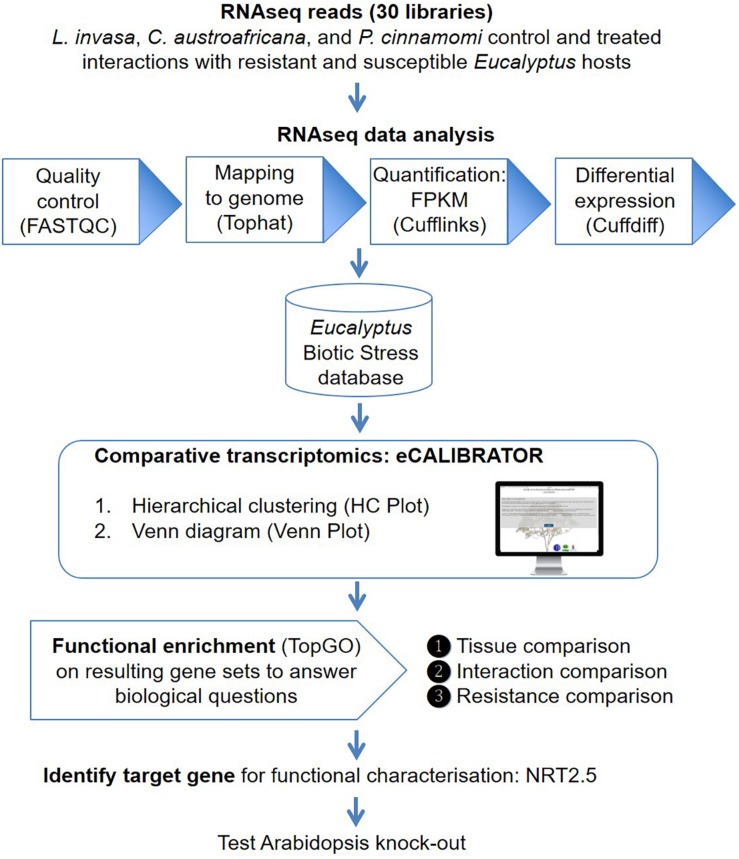
An overview of the workflow. Thirty RNA-seq libraries were analyzed with the Tuxedo suite. Gene and transcript expression values were imported into the Eucalyptus biotic stress database, a MySQL relational database, that also keeps track of the experiment and sample information. The HC Plot and Venn Plot tools from eCALIBRATOR was used to carry out a comparative transcriptomics analysis and gene lists were identified to answer specific biological questions. Resulting gene lists were subject to gene ontology (GO) enrichment analysis and a target gene, NITRATE TRANSPORTER 2.5 (NRT2.5), common among responses between different Eucalyptus-biotic stress interactions and linked to an enriched GO-term for this gene list was identified. Functional characterization in Arabidopsis supported a direct role for NRT2.5 in plant defense.

**FIGURE 2:**
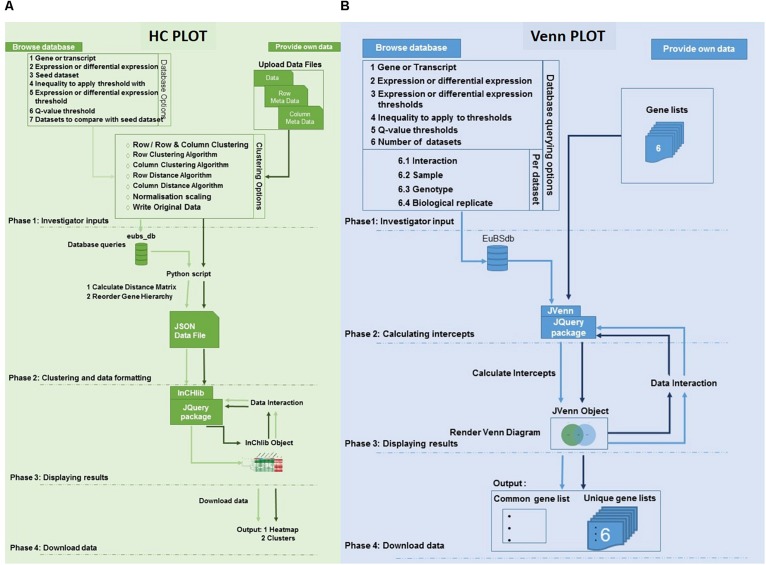
Architecture of the eCALIBRATOR tool. **(A)** The flow of the HC Plot pipeline for the “PROVIDE OWN DATA” option is in dark green while the flow of the pipeline for the “BROWSE DATABASE” option is in light green. The pipeline is split into five different phases. PHASE 1: Investigator options specific to the data input method, PHASE 2: Clustering and data formatting, PHASE 3: Displaying results and PHASE 4: Download data. **(B)** The flow of the Venn Plot pipeline for the “PROVIDE OWN DATA” option is in dark blue while the flow of the pipeline for the “BROWSE DATABASE” option is in light blue. The pipeline is split into four different phases. PHASE 1: Investigator options specific to the data input method, PHASE 2: Calculating the intercepts required to generate the venn, PHASE 3: Displaying results and PHASE 4: Download data.

The eCALIBRATOR (see text footnote 1) web-based tool consists of two sub-tools namely HC Plot and Venn Plot. The HC Plot tool exposes sets of genes or transcripts that show a similar pattern of expression or differential expression, across several experiments. The Venn Plot tool compares lists of genes or transcripts from a set of experiments, and identifies the genes or transcripts that are shared between the experiments or unique to an experiment. Both tools are comparative and result in an interactive visual output, which allows the investigator to interact with the output and the underlying data. Altering the data, as well as certain components of the visual output, will update the visual output itself. The visual output for the HCPlot is a heatmap with dendrogram; while the visual output for the VennPlot tool is a Venn or Edward’s diagram. Figure 1 outlines the workflow and how HC Plot and Venn Plot can be used to find key gene targets to further functionally characterize. Figure 2 shows simplified forms of the two pipelines.

For the HC Plot tool (Figure 2A), data can be retrieved from the Eucalyptus biotic stress database, through the “BROWSE DATABASE” option or data can be supplied by the investigator through the “PROVIDE OWN DATA” option, in which case the investigator is required to upload a data file as well as corresponding meta-data files. The meta-data files are used to annotate the rows and columns of the data file with additional information. When selecting the “BROWSE DATABASE” option, a few additional options are required for example: a seed dataset needs to be selected, a choice between whether expression data or differential expression data should be retrieved, an inequality and expression threshold as well as a Benjamini and Hochberg *q*-value threshold for multiple test correction (calculated by Cuffdiff v2.0) needs to be selected. An appropriate default value is given for all filtering thresholds.

**FIGURE 3:**
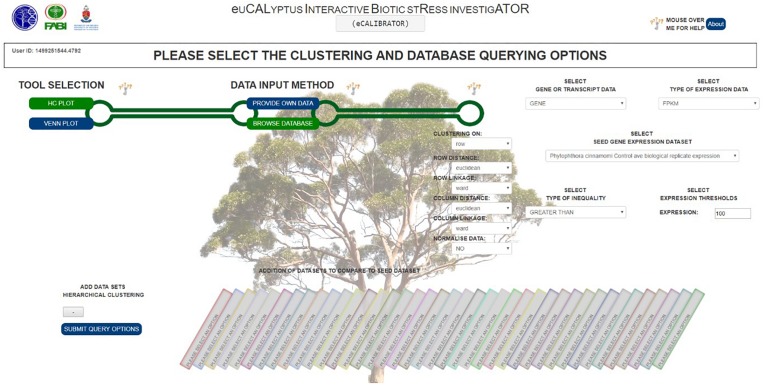
The HCPlot ‘BROWSE DATABASE’ options selection page.

Furthermore, a few clustering options can be selected (Figure 3): (1) Clustering on rows only or clustering on both rows and columns; (2) The distance and linkage algorithms for row and column clustering; (3) Data normalization (typically if the data provided has different metric scales for the individual experiments); selecting “yes” for this option scales all data so that values range from 0 to 1; and (4) Adding the expression values (original or normalized values) to the visual output; selecting “yes” will display the values. After submitting the relevant clustering options and file locations to a Python script, clustering (a two-step process) can be performed. The first step is calculating the distance between genes or transcripts (rows) and experiments or experimental groups (columns), based on the chosen metric. The second step is to reorder the rows and columns, based on the linkage metric chosen.

**FIGURE 4:**
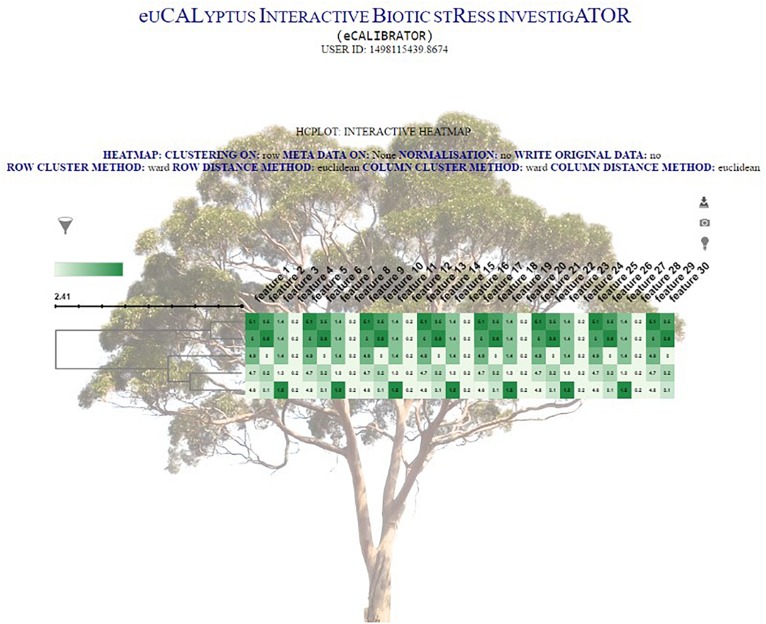
The HCPlot results page.

The clustering output is then formatted into JavaScript Object Notation (JSON) format. This JSON file is then passed to a JQuery plug-in, called InCHlib (Škuta et al., 2014), where the InCHlib object or heatmap is rendered and displayed on the results page. The investigator can view and interact with the clustered heatmap (Figure 4). Upon interaction, the heatmap is altered, re-rendered and a new heatmap is displayed. It is possible to download the heatmap as a publication quality image (.SVG or .PNG) or download a data file (.csv) containing the closest terminal nodes of the clustering process.

**FIGURE 5:**
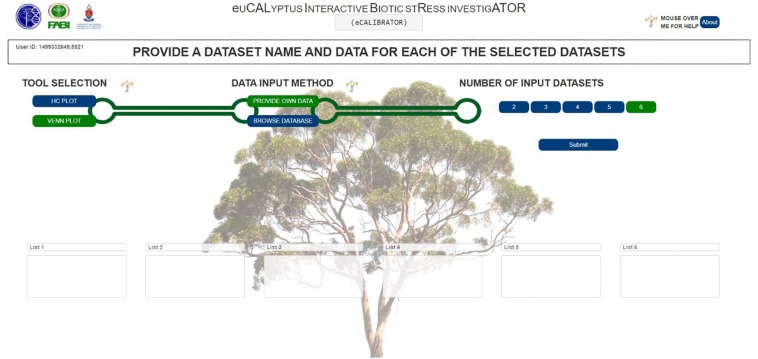
The VennPlot ‘PROVIDE OWN DATA’ options selection page.

**FIGURE 6:**
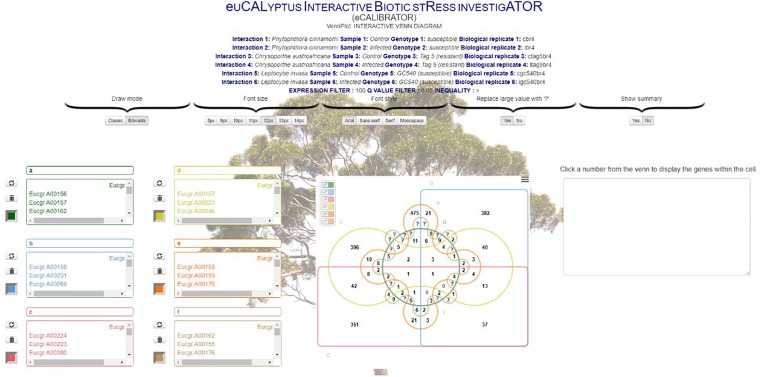
The VennPlot Edwards diagram page.

For the VennPlot tool (Figure 2B), data can also be retrieved from the Eucalyptus biotic stress database, through the “BROWSE DATABASE” option or data can be supplied by the investigator through the “PROVIDE OWN DATA” option, in which case the user is provided with a place to input a name, and paste a list, for each of the datasets chosen (Figure 5). The names and lists are then passed to the JVenn JQuery plugin (Bardou et al., 2014), when the investigator clicks the “Submit” button. The JVenn plugin then calculates a number of intercepts between the supplied datasets and uses them to create a JVenn object which can take the form of either a Venn or Edward’s diagram (Figure 6).

The investigator may also choose the “BROWSE DATABASE” option where the investigator makes choices with which to query the Eucalyptus biotic stress database. Between two and six datasets can be selected. The investigator will be asked to input a name, and select an interacting pathogen, a sample, a genotype and a biological replicate for each of the datasets. The investigator needs to choose whether to retrieve gene or transcripts data, as well as expression or differential expression data. The investigator then chooses an inequality and a threshold for the type of data chosen as well as a Benjamini and Hochberg *q*-value threshold for multiple testing correction (calculated by Cuffdiff v2.0).

The results page allows the investigator to interact with the Venn or Edward’s diagram. As with the HCPlot tool, an alteration of the data, or switching between the Venn and Edward’s diagrams, causes a re-calculation of the intercepts, and a new Venn or Edwards diagram to be produced. Once the investigator is satisfied with the results, the investigator can download a data file for the lists in the form of a comma-separated values (CSV) file, and a portable network graphic (PNG) or scalable vector graphics (SVG) file of the visual output.

### Comparisons and Gene Ontology Enrichments

We looked at tissue specific expression and noted that terms related to “RNA methylation” was present in the set of stem tissue enrichment terms (data not shown). Plants are known to utilize gene silencing through interfering RNA molecules such as miRNA as a strategy to overcome pathogen virulence, by silencing pathogenic RNAs.

**FIGURE 7:**
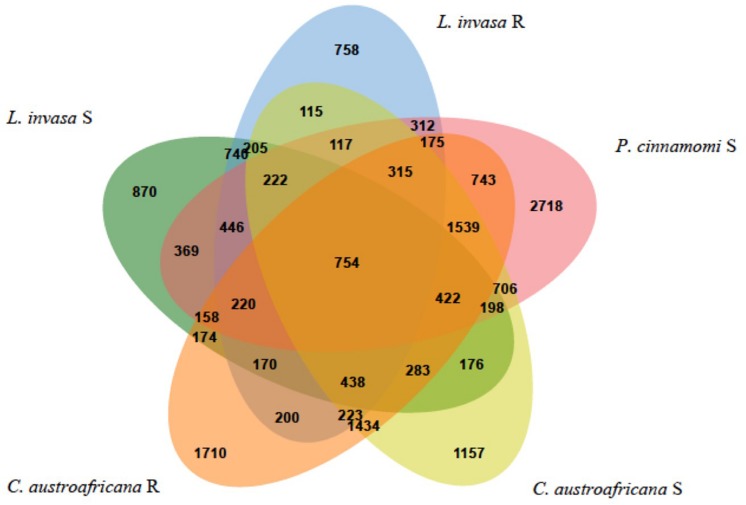
Comparisons of resistant and susceptible defense responses occurring in three biotic stress interactions. Results were generated using the Venn Tool in eCALIBRATOR and data is derived from Eucalyptus species challenges with the susceptible and resistant interactions of the stem canker pathogen, *Chrysoporthe austroafricana*, the insect pest, *Leptocybe invasa* and oomycete pathogen, *Phytophthora cinnamomi*.

The five interactions with Eucalyptus were split into the two resistant and three susceptible interactions, to answer the questions: “Which differentially expressed genes are in common between resistant and susceptible interactions?” and “Which differentially expressed genes are unique to either the resistant or susceptible?”. Figure 7 shows the results of the comparison between the five interactions. The interactions with *P. cinnamomi* Susceptible (S), *C. austroafricana S* and *C. austroafricana* Resistant (R) had the highest number of differentially expressed genes, while *L. invasa* S and *L. invasa* R had the least. The set of differentially expressed genes that were shared between the resistant and susceptible interactions with *C. austroafricana* and *P. cinnamomi* and the set of differentially expressed genes shared between resistant and susceptible *C. austroafricana* were similar in the number of differentially expressed genes as the unique sets.

### Hierarchical Clustering and Functional Enrichment of Differentially Expressed Genes Common Between Resistant and Susceptible Interactions

**FIGURE 8:**
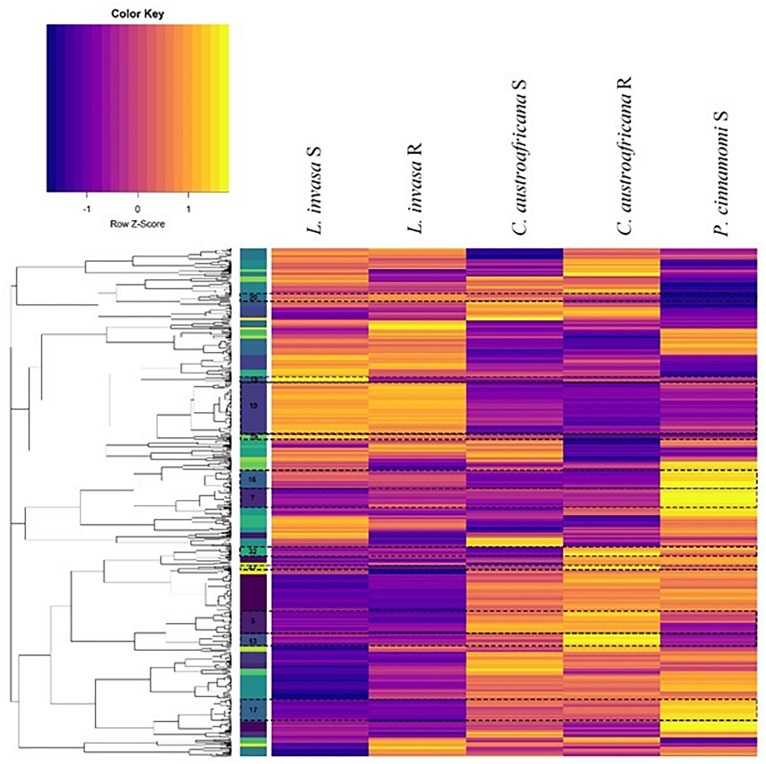
Hierarchical clustering of differentially expressed genes common to resistant and susceptible defense responses. Columns represent interactions with Eucalyptus while rows represent genes. The colored bars to the left of the heatmap represent individual clusters. Eleven clusters (highlighted and marked with a cluster number) have significant enrichment of biological process gene ontology terms. The purple color in the heatmap represents genes that are down-regulated while yellow represents genes that are up-regulated.

The set of differentially expressed genes in common between the two resistant and the three susceptible interactions (754) were put through hierarchical clustering to identify genes that are regulated to the same degree. Hierarchical clustering resulted in 52 distinct clusters, which can be seen in Figure 8. The colored bar with numbers in-between the dendrogram and heatmap (in Figure 8) shows each of the 52 clusters as unique colors. Each cluster also shows a distinct regulation pattern in each of the five interactions where purple represents down-regulation and yellow represents up-regulation.

Each cluster consisted of between 3 and 77 differentially expressed genes. Only 11 clusters (5, 7, 10, 13, 15, 16, 17, 20, 32, 39, and 40; marked in Figure 8) had significant enrichment. Supplementary Table S2 shows the non-redundant enriched BP associated with the set of differentially expressed genes shared between resistant and susceptible interactions. The term “lignin catabolic process” was the only enriched term from cluster 5. All four genes identified under this term were laccases, three of which were homologs. These genes were up-regulated in resistant interactions and were down-regulated in the susceptible interactions. Cluster 7 was enriched with the term “cellular water homeostasis” which is a known process involved with wounding. All genes under this term were wall associated kinase 2 homologs. Also, genes enriched in this term were up-regulated in resistant responses while being down regulated within susceptible responses. Cluster 15 was enriched with the term “negative regulation of ethylene-activated signaling pathways.” This term is up-regulated in susceptible interactions, and down-regulated in the resistant interactions. Cluster 16 contained multiple terms related to plant defense such as “response to hydrogen peroxide” as well as two more terms which are “response to chitin” and “detection of biotic stimulus.” All three of these terms were down-regulated in resistant and up-regulated in the susceptible interactions. This cluster also had a considerable amount of terms (in the redundant list; data not shown) associated with nitrogen metabolism all of which were down-regulated in the resistant interaction and up-regulated in the susceptible. Cluster 17 had another term relating to nutrients, which was “regulation of response to nutrient levels,” which had a similar pattern of expression. Cluster 32 had enrichment for several defense related terms associated with defense against insects. These terms included the “regulation of defense response to insects” and “indole-glucosinolate biosynthetic process.” Two genes that were enriched under this term are DNA-binding proteins involved in RNA-regulation of transcription and were up-regulated in all resistant and susceptible interactions. These two genes were also the only genes enriched in another three terms in cluster 32. All terms enriched in Cluster 32 were up-regulated in the resistant interactions, while being down-regulated in the susceptible interactions. Cluster 39 had the single term, “phototropism” enriched, which was down-regulated in the resistant interactions, but up-regulated in the susceptible-interactions.

### Functional Enrichment of Differentially Expressed Genes Unique to Either Resistant or Susceptible Interactions

Enriched terms identified in the unique sets of differentially expressed genes for resistant and susceptible interactions is presented in Supplementary Table S3. All except the *C. austroafricana* susceptible up-regulated datasets had enriched terms identified in the unique sets of differentially expressed genes for resistant and susceptible interactions (Supplementary Table S3). A large majority of the terms enriched in the unique sets (except for the susceptible *P. cinnamomi* down-regulated set), are associated with the regulation of normal metabolic related processes.

The set of enriched terms in the resistant down-regulated *C. austroafricana* interaction are not directly involved with plant defense, however, may contribute to the regulation of it. The first term “protein import into the peroxisome matrix,” while the second term enriched in this set is “fatty acid-beta oxidation.” The presence of such terms in other resistant defense responses was not observed. The up-regulated resistant *C. austroafricana* terms was “photorespiration.” Terms such as “glycerolipid metabolic process” and “lipid biosynthetic process,” enriched in resistant up-regulated *L. invasa* interaction, indicate that triglyceride biosynthesis may be used to synthesize lipids. The down-regulated terms enriched for the unique *L. invasa* set indicated that growth and developmental processes associated with leaf tissues, in general were down-regulated. Surprisingly, there were no up-regulated terms for the susceptible host response to *C. austroafricana*. Enrichment in the down-regulated susceptible host response to *L. invasa*, similar to the down-regulated host response to *C. austroafricana*, show a down-regulation of leaf-tissue specific metabolisms. Terms included “chloroplast relocation,” “transcription from plastid promoter,” and “thylakoid membrane organization.” Terms observed in the susceptible up-regulated response to the *P. cinnamomi* interaction included, “respiratory burst involved in defense response,” “regulation of plant-type hypersensitive response,” and “regulation of hydrogen peroxide metabolic process.” For the susceptible down-regulated response in the *P. cinnamomi* interaction, a group of enriched terms relating to growth and development was observed. Such terms include “root hair elongation,” “plant-type secondary cell wall biogenesis,” and “multidimensional cell growth.” The term “response to organonitrogen compound,” “response to nitrogen compound” and “regulation of response to nutrient levels” were observed within the resistant interactions. Among the latter enriched GO terms was the *Eucgr.H02533.1* gene, annotated as a nitrate transporter. This gene, was particularly interesting because it was up-regulated in susceptible and down-regulated in moderately resistant interactions.

### Functional Genetics Analysis of AtNRT2.5 in Plant Defense

**FIGURE 9:**
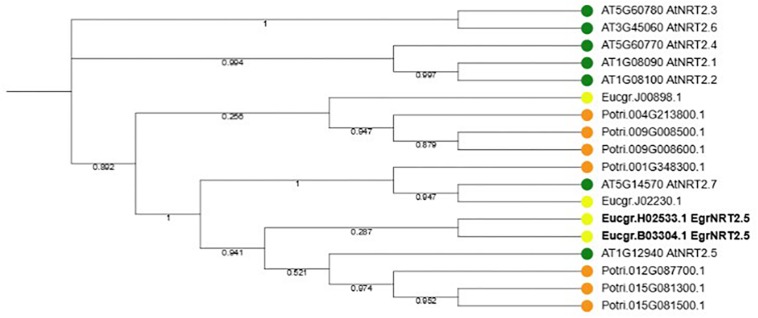
Phylogenetic tree of NRT2.5 and family members in *Arabidopsis thaliana, Eucalyptus grandis* and *Populus trichocarpa*. Seven NRT2 genes occur in *A. thaliana* and *P. trichocarpa* whereas four NRT2 genes occur in *E. grandis*. Two *E. grandis* NRT2.5 orthologs, EucgrH02533.1 and EucgrB03304.1 are highlighted in bold font.

According to the phylogenetic tree, the ortholog of the Eucalyptus gene Eucgr.H02533.1 is AtNRT2.5 (Figure 9). The role of AtNRT2.5 has been demonstrated in nitrate acquisition and remobilization under long-term nitrate starvation in Arabidopsis (Lezhneva et al., 2014), however, the functional role of this protein in plant defense is unclear. The importance of AtNRT2.5 in plant defense was evaluated using T-DNA insertion mutants. Two independent homozygous T-DNA insertion lines were identified, GABI-Kat 213H10 (atnrt2.5-A) and GABI-Kat 046H04 (atnrt2.5-B) by selection on MS plates containing sulfadiazine and PCR. The T-DNA was detected in atnrt2.5-A and atnrt2.5-B mutants using a combination of the T-DNA left border oligonucleotide and gene-specific oligonucleotide by the presence of an 800 and 550 bp fragment, respectively (Supplementary Figure S1). The absence of a 1243 and 980 bp fragment in the atnrt2.5-A and atnrt2.5-B mutants, respectively, using forward and reverse gene-specific oligonucleotides spanning the T-DNA insertion site validated atnrt2.5-A and atnrt2.5-B as homozygous T-DNA insertion lines (Supplementary Figure S1). RT-PCR also verified that in both mutants, AtNRT2.5 expression was absent (results not shown), establishing atnrt2.5-A and atnrt2.5-B as homozygous knockout lines.

### Disruption of AtNRT2.5 Results in Reduced Susceptibility to *Pseudomonas syringae* pv. *tomato* DC3000

**FIGURE 10:**
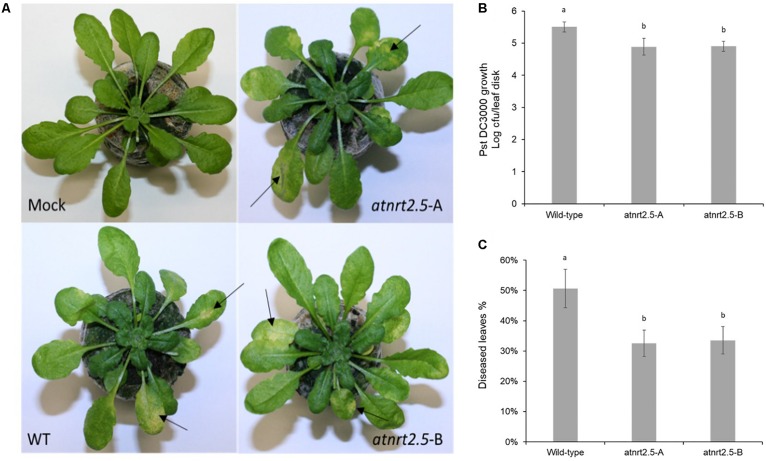
Symptom development in wildtype (WT) and nrt2.5 mutant plants following infection with *Pst*DC3000. **(A)** Representative examples of chlorosis development, **(B)** bacterial proliferation and **(C)** percentage of diseased leaves. Error bars represent the standard error of the mean of six plants. Different letters represent significant differences based on student’s *T*-test (*p* < 0.01).

To test whether AtNRT2.5 is required for plant defense, normally fertilized 5-week-old WT Col-0 and atnrt2.5 plants were inoculated by dipping in a suspension of Pst DC3000. As evidenced by bacterial proliferation, both atnrt2.5 mutants, atnrt2.5-A and atnrt2.5-B, showed reduced susceptibility to the pathogen 24 hpi as compared to the wildtype (Figure 10B). Both mutants also showed reduced symptom severity 3-days post inoculation (Figure 10C). Symptoms on jiffy^TM^ and hydroponically grown Arabidopsis plants included water-soaking which appeared ∼48 h post inoculation, and necrosis and chlorosis which appeared ∼72 h post inoculation (Figure 10A).

## Discussion

### The eCALIBRATOR Tool

We developed the eCALIBRATOR tool, which consists of two comparative tools – A Venn Tool (Venn Plot) and a clustering tool (HC Plot). Both tools are interactive and provide access to the gene-lists contained within the visual outputs of the tools. Both tools allow data input by two methods, either by data submitted by the user, or by accessing data directly from the relational database Eucalyptus biotic stress database.

### *Eucalyptus* Defenses

eCALIBRATOR was also developed to address questions regarding the Eucalyptus defense response, focusing on tissue-specific responses, biotic stress-specific and shared responses, resistance or susceptibility signatures, and to identify some core defense pathways to target for functional genetic testing.

### Shared Responses

The main shared responses of Eucalyptus to the interactions with *L. invasa*, *C. austroafricana*, and *P. cinnamomi* show a diversity of known defense responses. The first of which was enrichment of terms relating to SAR. This indicates that Eucalyptus is capable of producing a systemic response from a diverse range of biotic challenges, which occur in both the leaf and stem tissues. Other enriched terms relating to SAR show that many of the associated preceding responses were also detected, which included the generation of ROS, the oxidative burst, and the hypersensitive response (Durrant and Dong, 2004).

Signaling is another fundamental aspect which controls the outcome of a defense response. Signaling in the form of phytohormones is complex, and cross-talk between the different signaling pathways is a mechanism which allows the host to fine-tune the defense response, based on the interaction (Pieterse et al., 2012). We observed the enrichment of several phytohormone terms known to be involved with Eucalyptus defense signaling. These hormones included SA, which is known to be involved in inducing SAR, and enrichment of the biological process “systemic acquired resistance, salicylic acid mediated signaling pathway” directly implicates it here. The other enriched hormones included abscisic acid, which has been linked to responses to *P. cinnamomi* (Meyer et al., 2016) and *C. austroafricana* (Mangwanda et al., 2015).

### Tailored Responses

Tailored responses in this study are concordant with results previously generated. The enriched biological processes that support previous enrichments in the *L. invasa* interaction were: the up regulation of “response to UV-B”; and “response to sucrose” (Oates et al., 2015). The role of carbohydrates is well established in plant defense, and are considered as constituents of a complex communication system, which is involved in coordinating metabolism and responses to stress (Rolland et al., 2006). Carbohydrate sprays have even been considered as potential primers of plant defense systems, as they are considered to be elicitors (Bolouri Moghaddam and Van den Ende, 2012; Bolouri Moghaddam and Van den Ende, 2013). In the *P. cinnamomi* interaction enrichment of “SAR,” “response to ethylene” and “JA mediated signaling pathway” support the enrichments conducted previously (Meyer et al., 2016). While the enrichments reported for the *C. austroafricana* interaction, did not share much similarity to previously reported enrichments (Mangwanda et al., 2015). This is possibly due to the large amount of terms enriched in the shared responses, which we identified with the elim method and newer annotation of the genome, while Mangwanda et al. (2015) based their GO universe on Arabidopsis identifiers.

### Resistant Signatures

Plant disease resistance is based on a stable inherited genetic ability to overcome factors of pathogenicity produced by a pathogen (Barnett, 1959; Van der Biezen and Jones, 1998). In order for the host to prevent pathogen progression and ultimately prevent disease it must first detect the threat (Zipfel, 2008), and secondly have the genetic repertoire to produce an appropriate response that thus limits pathogen progression (Zhang et al., 2013). Various signals were detected in this study that suggest that Eucalyptus is able to achieve this. The first of these related to threat detection, the term “detection of biotic stimulus” along with “response to chitin” was apparent in resistant interactions. The “response to chitin” was found in the up-regulated gene set in the resistant interactions (interactions with *L. invasa* and *C. austroafricana*). It is well known that chitin is present in insects and fungi, however, is absent in oomycetes, where cellulose is found instead (Mach, 2008; Petutschnig et al., 2014; Zhu et al., 2016).

In two of the comparisons conducted in this study, terms relating to nitrogen have appeared, making it a common theme, and highlights the importance of nutrients in Eucalyptus disease resistance. In the interaction comparison, enrichment of the term “response to organonitrogen compound” and “response to nitrogen compound” in the shared enrichment set were observed. In the resistance comparison, the terms “response to organonitrogen compound,” “response to nitrogen compound” and “regulation of response to nutrient levels” also appear in the shared set of enrichments. Most pathogens are effectively nutrient starved when in first contact with the host plant, this makes the acquisition of nutrients by the pathogen essential for pathogen survival (Mur et al., 2017). Further, there are two opposing views on nitrogen levels which directly impact the success of pathogens, which have been suggested by Mur et al. (2017). The first view suggests that nitrogen is a limited nutrient, while the second view suggests it is abundant. It is also suggested that nitrogen starvation may play a role in controlling the expression of pathogenicity genes (Thomma et al., 2006; López-Berges et al., 2010). The *Eucgr.H02533.1* gene, annotated as a nitrate transporter, was present among these enriched GO terms. In particular, this gene was up-regulated in susceptible and down-regulated in moderately resistant interactions. These profiles suggest that possibly the pathogens may be manipulating the host for nitrate through an up-regulation of Eucgr.H02533.1 leading to a susceptible interaction. The host may be trying to counteract this manipulation by down-regulating *Eucgr.H02533.1*, leading to a resistant interaction.

### AtNRT2.5 Is Involved in Defense Against Pathogens

A phylogenetic comparison of the *Eucgr.H02533.1* gene confirmed that the gene is orthologous to *AtNRT2.5*, a nitrate transporter. The functional role of this gene was described by Lezhneva et al. (2014) in nitrate acquisition and remobilization under long-term nitrate starvation in Arabidopsis; however, its role in defense was not determined. In *Arabidopsis*, *AtNRT2.5* was up-regulated in the leaves during interactions with the bacterial symbiont *Phyllobacterium brassicacearum* strain STM196 and the endophytic fungus *Piriformospora indica* (Kechid et al., 2013; Vahabi et al., 2015). Furthermore, *AtNRT2.5* was up-regulated when challenged with the hemibiotrophic pathogen *Pseudomonas syringae* pv. *tomato* DC3000 and elicitors, e.g., flagellin 22 oligopeptide (flg22). The aforementioned studies demonstrate the importance of NRT2.5 in plant-pathogen interactions yet to date, the functional role of this protein in plant defense is unclear. We validated the role of AtNRT2.5 in biotic stress response using the Arabidopsis-*Pst* DC3000 interaction. Fertilizing Arabidopsis plants with 0.5 mM or more ammonium nitrate activates the low-affinity transport system and suppresses the high-Affinity transport system which includes the repression of AtNRT2.5 (Lezhneva et al., 2014). Therefore, to investigate the role of AtNRT2.5 in plant defense we performed experiments with optimal fertilization of 7 mM nitrate. In this condition, we show that two independent T-DNA knockouts of AtNRT2.5 have reduced susceptibility to the bacterial pathogen. This finding is consistent with the result obtained by Camañes et al. (2012) that showed that atnrt2.1 mutants are less susceptible to Pst DC3000. This suggests AtNRT2.1 and AtNRT2.5 may have overlapping mechanisms in defense under *Pst* DC3000 challenge; however, this hypothesis remains to be confirmed. Based on these findings, in the susceptible interaction in *Eucalyptus*, up-regulation of *Eucgr.H02533.1* may lead to a repression of a defense pathway e.g., the salicylic acid defense pathway, to promote susceptibility. In the resistant interaction, down-regulation of *Eucgr.H02533.1* may activate the same defense pathway to promote resistance.

## Conclusion

The tools available within eCALIBRATOR facilitated powerful comparative transcriptomic analyses, that allowed investigation into Eucalyptus defense responses, to reveal shared and tailored defense responses to different perturbations. Terms “response to organonitrogen compound” and “response to nitrogen compound” emerged as important gene ontology enrichments with the *E. grandis* ortholog of *NRT2.5* gene as a signature gene. The Arabidopsis *NRT2.5* was shown for the first time to be involved in plant defense. The tool can be extended to other species for which transcriptomic data is available, allowing for further insight into cross-species conservation of gene function in plant defense.

## Data Availability Statement

All datasets generated for this study are included in the article/Supplementary Material.

## Author Contributions

SN and NC conceived the study. YdT developed eCALIBRATOR and DC conducted all the functional genetics analysis together with RM. RM helped interpret the results of the mutant analysis. SN and NC wrote the manuscript with input from YdT, DC, and RM.

## Conflict of Interest

The authors declare that the research was conducted in the absence of any commercial or financial relationships that could be construed as a potential conflict of interest.

## Footnotes

1 http://phylo.bio.ku.edu/software/sate/sate.html

2 trimal.cgenomics.org/

3 https://winscp.net/eng/download.php

4 http://www.chiark.greenend.org.uk/∼sgtatham/putty/download.html

## Supplementary Material

FIGURE S1 Selection of atnrt2.5 T-DNA mutants. **(A)** Diagram of the AtNRT2.5 gene consisting of three exons and two introns with the positions of the T-DNA insertions in GK213H10 (AtNRT2.5-A) and GK046H04 (AtNRT2.5-B) in the second and first exons, respectively. **(B,C)** PCR detection of T-DNA in AtNRT2.5-A **(B)** and AtNRT2.5-B **(C)**, respectively, using a combination of the T-DNA left border oligonucleotide and gene-specific oligonucleotides. No amplification was observed in AtNRT2.5-A and AtNRT2.5-B using gene-specific oligonucleotides spanning the T-DNA insertion sites.

TABLE S1 Summary of the mapping of RNA-seq libraries per Eucalyptus–pathogen interaction sample.

TABLE S2 Functionally enriched terms of the gene ontology biological processes category in common between the resistant and susceptible defense responses.

TABLE S3 Functionally enriched terms of the gene ontology biological processes category in the unique set of differentially expressed genes between the resistant and susceptible interactions.
