# Supplementary figures and images for: eCALIBRATOR: A Comparative Tool to Identify Key Genes and Pathways for Eucalyptus Defense Against Biotic Stressors
Source: Front Microbiol. 2020 Feb 17;11:216. doi: 10.3389/fmicb.2020.00216 (PMC7039109; doi:10.3389/fmicb.2020.00216)

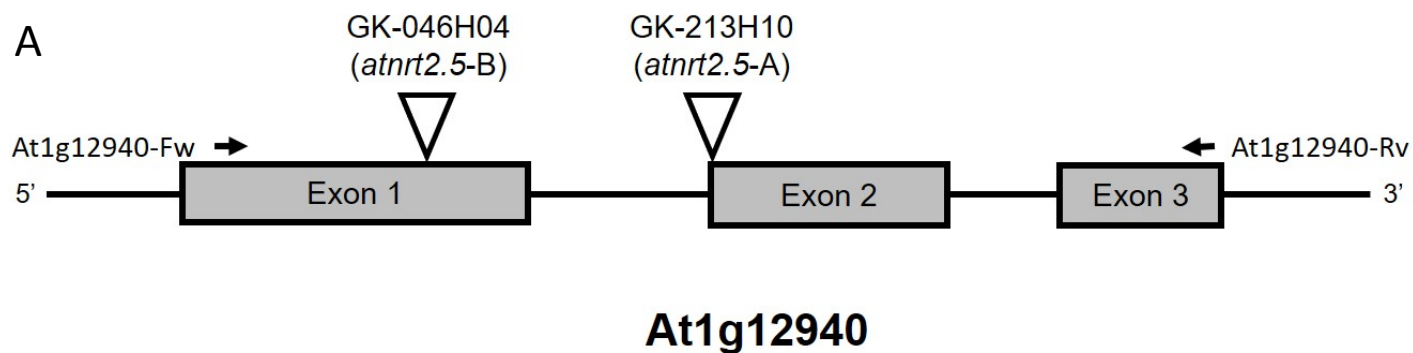

**B**

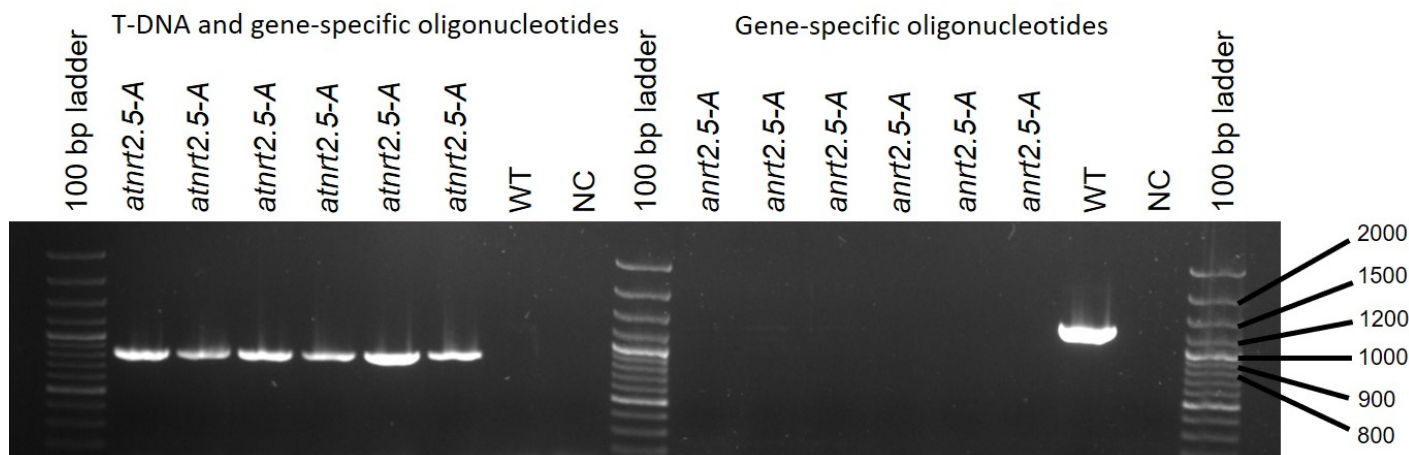

**C**

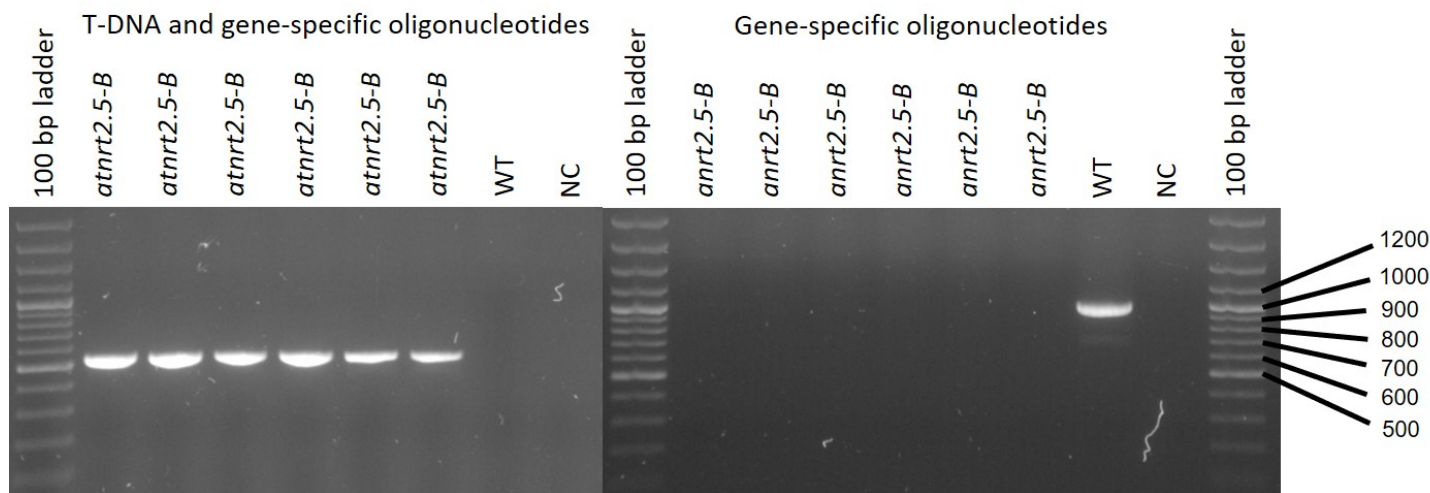

Supplement: FIGURE S1 — Selection of atnrt2.5 T-DNA mutants. (A) Diagram of the AtNRT2.5 gene consisting of three exons and two introns with the positions of the T-DNA insertions in GK213H10 (AtNRT2.5-A) and GK046H04 (AtNRT2.5-B) in the second and first exons, respectively. (B,C) PCR detection of T-DNA in AtNRT2.5-A (B) and AtNRT2.5-B (C), respectively, using a combination of the T-DNA left border oligonucleotide and gene-specific oligonucleotides. No amplification was observed in AtNRT2.5-A and AtNRT2.5-B using gene-specific oligonucleotides spanning the T-DNA insertion sites. [file Image_1.pdf]
